# Splanchnic sympathetic nerve denervation improves bacterial clearance and clinical recovery in established ovine Gram-negative bacteremia

**DOI:** 10.1186/s40635-023-00530-6

**Published:** 2023-08-03

**Authors:** Rachel M. Peiris, Clive N. May, Lindsea C. Booth, Robin M. McAllen, Michael J. McKinley, Sally Hood, Davide Martelli, Rinaldo Bellomo, Yugeesh R. Lankadeva

**Affiliations:** 1 Preclinical Critical Care Unit, Florey Institute of Neuroscience and Mental Health, University of Melbourne, 30 Royal Parade Parkville, Victoria, 3052 Australia; 2 Department of Critical Care, Melbourne Medical School, University of Melbourne, Victoria, Australia; 3 Department of Biomedical and Neuromotor Sciences, University of Bologna, Bologna, Italy; 4 Department of Intensive Care, Austin Health, Victoria, Australia; 5 Australian and Intensive Care Research Centre, Monash University, Melbourne, Australia

**Keywords:** Bacteremia, Sepsis, Splanchnic sympathetic nerves, Cytokines, Inflammation

## Abstract

**Background:**

The autonomic nervous system can modulate the innate immune responses to bacterial infections via the splanchnic sympathetic nerves. Here, we aimed to determine the effects of bilateral splanchnic sympathetic nerve denervation on blood pressure, plasma cytokines, blood bacterial counts and the clinical state in sheep with established bacteremia.

**Methods:**

Conscious Merino ewes received an intravenous infusion of *Escherichia coli* for 30 h (1 × 10^9^ colony forming units/mL/h) to induce bacteremia. At 24 h, sheep were randomized to have bilaterally surgically implanted snares pulled to induce splanchnic denervation (N = 10), or not pulled (sham; N = 9).

**Results:**

Splanchnic denervation did not affect mean arterial pressure (84 ± 3 vs. 84 ± 4 mmHg, mean ± SEM; P_Group_ = 0.7) compared with sham treatment at 30-h of bacteremia. Splanchnic denervation increased the plasma levels of the pro-inflammatory cytokine interleukin-6 (9.2 ± 2.5 vs. 3.8 ± 0.3 ng/mL, P_Group_ = 0.031) at 25-h and reduced blood bacterial counts (2.31 ± 0.45 vs. 3.45 ± 0.11 log10 [CFU/mL + 1], P_Group_ = 0.027) at 26-h compared with sham treatment. Plasma interleukin-6 and blood bacterial counts returned to sham levels by 30-h. There were no differences in the number of bacteria present within the liver (P_Group_ = 0.3). However, there was a sustained improvement in clinical status, characterized by reduced respiratory rate (P_Group_ = 0.024) and increased cumulative water consumption (P_Group_ = 0.008) in splanchnic denervation compared with sham treatment.

**Conclusion:**

In experimental Gram-negative bacteremia, interrupting splanchnic sympathetic nerve activity increased plasma interleukin-6, accelerated bacterial clearance, and improved clinical state without inducing hypotension. These findings suggest that splanchnic neural manipulation is a potential target for pharmacological or non-pharmacological interventions.

**Supplementary Information:**

The online version contains supplementary material available at 10.1186/s40635-023-00530-6.

## Background

A dysregulated immune response to bacterial infections can be a common trigger for sepsis [1, 2]. Reductions in blood pressure in response to systemic bacterial infections elicit a baroreflex-mediated increase in sympathetic nerve activity (SNA) [3, 4], which would be expected to promote peripheral vasoconstriction. However, this effect appears offset by the profoundly elevated release of vasodilators such as nitric oxide, leading to a net reduction in total peripheral conductance and mean arterial pressure (MAP). There is, however, accumulating evidence that protracted overactivation of the splanchnic sympathetic nerves can exacerbate the sepsis-associated immune suppression and promote bacterial growth [5–7]. Accordingly, developing interventions to modulate splanchnic SNA may provide a new therapeutic avenue for sepsis.

In response to systemic inflammation, we established that the brain activates a powerful anti-inflammatory reflex through the splanchnic sympathetic nerves [8]. In a rodent model of endotoxemia, we demonstrated that an anti-inflammatory reflex mediated by the splanchnic sympathetic nerves strongly suppresses tumor necrosis factor alpha (TNF-α) response, within 1.5-h of lipopolysaccharide treatment [8, 9]. In addition, we showed that sheep with previously cut splanchnic nerves (to disable the reflex) had an enhanced TNF-α response within 1.5-h and interleukin-6 (IL-6) response within 6-h of an intravenous bolus of live *Escherichia coli* (*E. coli)*. Such animals also cleared their bacteremia more rapidly [10]. However, implementing interventions prior to the onset of bacteremia lacks clinical translatability, because most patients treated in intensive care units have established infections [11, 12]. Additionally, the safety of denervating a major resistance vascular bed such as the splanchnic circulation in the late stages of bacterial infection remains unknown.

Accordingly, we aimed to assess the safety and effectiveness of splanchnic denervation after 24-h of a systemic Gram-negative infection in a well-validated sheep model [13]. We investigated whether splanchnic denervation was able to reverse immunosuppression, accelerate bacterial clearance and improve the clinical state when it was applied during established bacteremia rather than beforehand. As the liver is established as a vital organ involved in limiting the systemic dissemination of bacteria in response to infection [14], we also assessed whether splanchnic denervation accelerated the number of *E. coli* sequestrated in the liver. We tested the hypothesis that splanchnic sympathetic nerve denervation would increase plasma levels of proinflammatory cytokines, accelerate bacterial clearance, increase liver sequestration, and thereby aid clinical recovery in sheep with established bacteremia.

## Materials and methods

### Animal preparation

The experiments were performed in accordance with the National Health and Medical Research Council of Australia guidelines and approved by the Animal Ethics Committee of the Florey Institute of Neuroscience and Mental Health [Ethics ID: 20-040-FINMH]. All studies were performed in accordance with the Animal Research: Reporting of In Vivo Experiments (arrive) criteria [15]. Merino Ewes (35–45 kg) aged between 1.5–2.5 years were housed in individual metabolic cages with access to 800 g of oaten chaff and 6.5 L of water daily.

### Surgical preparation

Sheep underwent a preparatory surgical procedure under sterile conditions. General anesthesia was induced with 15 mg/kg of sodium thiopental (intravenous, [i.v.], Jurox Pty Limited, NSW, Australia) and following tracheal intubation, maintained on 2.0 – 2.5% isoflurane (Pharmachem, QLD, Australia) at an inspired oxygen fraction of 60%.

The splanchnic sympathetic nerves were bilaterally exposed retroperitoneally via flank incisions. Once a splanchnic sympathetic nerve was isolated, a silk snare was passed under each nerve (length 30 cm, size 3-O; Dynek PTY LTD, SA, Australia). Both ends of the snare were passed up a vinyl tube (length 20 cm, ID 1.5 mm, OD 2.5 mm; Dural Plastics & Engineering, NSW, Australia) and a polyethylene cannula (length 25 cm, ID 0.58 mm, OD 0.96 mm; Microtube Extrusions, NSW, Australia) was pushed down the vinyl tube so that its tip was adjacent to the nerve for the administration of an anesthetic agent prior to denervating the animals in the treatment group (Additional file 1: Fig. S1). An in-house fabricated thermocouple was implanted in the retroperitoneal space to measure core body temperature. The tubes encasing the snare and the cannula were tunneled subcutaneously and exteriorized on the back of the sheep.

Two polyethene cannulae were inserted into the right jugular vein for the administration of *E. coli* (ID 0.58 mm, OD 0.96 mm; Microtube Extrusions, NSW, Australia) and maintenance fluid infusions (ID 1.18 mm, OD 1.7 mm; Microtube Extrusions, NSW, Australia). A tygon cannula (ID 1.0 mm, OD 1.8 mm; Cole-Parmer, VIC, Australia) was inserted into the right carotid artery for blood sampling and measurement of MAP and heart rate (HR).

Animals were administered intramuscular antibiotics (penicillin, 900 mg Ilium, Troy Laboratories, VIC, Australia) under general anesthesia and then at 24- and 48-h postoperatively. Flunixin meglumine (50 mg, intramuscular; Flunixon, Norbrook, Tullamarine, Australia) was administered at surgery and then 4-h postoperatively for analgesia. Animals were allowed a 3-day recovery period before initiating the experimental protocol.

### Experimental protocol

#### Induction of Gram-negative bacteremia

**Fig. 1:**
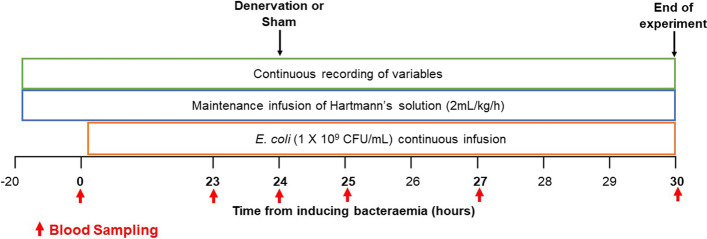
Experimental timeline from baseline to 30-h of Gram-negative bacteremia. Timepoint − 20 represents the 20 h of baseline recordings. Timepoint 0 indicates the 20th h of baseline recordings prior to starting the continuous infusion of *E. coli.* All animals were given a maintenance fluid infusion of Hartmann’s solution (-20 to 30-h from inducing bacteremia) and a continuous infusion of *E. coli* (0 to 30-h from inducing bacteremia). At 24-h of bacteremia animals underwent either splanchnic denervation or sham procedure. In bold are the sampling timepoints (0, 23, 24, 25, 27 and 30 from inducing Gram-negative bacteremia)

After a 20-h baseline period, bacteremia was induced by continuous intravenous infusion of *E. coli* for 30-h (1 X 10^9^ colony forming units [CFU]/mL, 0.6 mL/h). This dose of *E. coli* has been previously reported to induce a clinically relevant hemodynamic profile in Gram-negative bacteremia in sheep [18, 19]. Sheep were given a maintenance infusion of Hartmann’s solution (i.v., 2 mL/kg/h; Baxter Pharmaceutical Solutions LLC, NSW, Australia) from the end of the baseline period to the end of the experiment, which was limited to 30-h of bacteremia before the animal was euthanized (Fig. 1).

### Intervention

At 24-h of bacteraemia, conscious sheep were randomly allocated to either the splanchnic-denervated (n = 10) or sham-denervated (sham n = 9) group. Sheep allocated to the splanchnic-denervated group were given 4 mL of lignocaine (20 mg/mL, ilium, Troy Laboratories PTY Limited, NSW, Australia) via the implanted polyethylene cannula, to avert pain when the snares implanted on the right-flank were pulled. Five minutes later, the splanchnic nerve snares were bilaterally pulled and clamped. After 5-min, this splanchnic denervation process was repeated contralaterally on the left flank of the animals. Sheep allocated to the sham group were given 4 mL of saline and were left untreated for a similar period. There was no blood sampling during 24–25 h in both groups. In both groups the bacterial infusion was continued for a further 6 h (total 30-h), after which the animals were euthanized (pentobarbitone sodium, 100 mg/kg, i.v., Vibrac PTY Limited, NSW, Australia). At post-mortem, the effectiveness of splanchnic denervation was confirmed, and sections of the liver were collected for immunohistochemical analysis of *E. coli* localization (Fig. 1).

### Sampling and recording

Arterial blood samples were collected at the 20th h of baseline, 23rd h of bacteremia (before splanchnic-denervation or sham procedure) and then after intervention at the 25th, 27th and 30th h of bacteremia. Cumulative food and water consumption and respiratory rate were measured during the 20th h of baseline, at 23-h of bacteremia and then at hourly intervals after splanchnic-denervation or sham treatment interventions (from 24-to-30-h of bacteremia) to assess clinical state. MAP, HR, and core body temperature were continuously recorded at 100 Hz on a computer using a CED micro 1401 interface and Spike2 software (Cambridge Electronic Design, Cambridge, United Kingdom).

### Immunohistochemical analysis

At post-mortem, sections of liver (approximately 5–6 g) were collected and fixed in 10% neutral buffered formalin. Samples were then dehydrated, paraffin embedded and 5 µm slices slide mounted, as previously described (Histology Core Services, Florey Institute) [16]. Slides were deparaffinized using xylene, cleared in graded alcohols, and rehydrated in 0.1 M PBS. Sections were stained using a rabbit anti-*E. coli* primary antibody (Abcam, Cambridge, United Kingdom; 1:500) at room temperature overnight. After washes, sections were then incubated in a Biotin-tagged anti-rabbit antibody (Jackson ImmunoResearch; 1:500) for 1-h at room temperature followed by Streptavidin-Alexa Fluor®488 conjugated secondary antibody (Jackson ImmunoResearch; 1:500) for 1-h at room temperature. Tissues were coverslipped with mounting medium containing DAPI (Sigma-Aldrich). Images were obtained using a Leica DMI6000 inverted confocal microscope at × 20 magnification. For each liver section six images were taken: three around hepatic vessels and three in the liver parenchyma between major vessels. *E. coli* were viewed using the Leica LasX software and then counted manually. The investigators undertaking the *E. coli* counting were blinded to the treatment groups.

### Cytokine analysis

Arterial plasma levels of IL-6, IL-8, IL-10, interferon-gamma (IFN-γ), and TNF-α were analysed using manufacturer validated enzyme-linked immunosorbent ovine assays (ELISA) (Kingfisher Biotech Inc., MN, USA) in accordance with the manufacturer’s guidelines (refer to Additional file 1: Table S1), as previously described [10].

All ELISA kits followed the same protocol, incubation with the capture antibody (2.5 µg/mL) and blocking buffer (4% BSA in DPBS). Arterial blood samples were collected in 5 mL EDTA tubes (Sarstedt, Germany), centrifuged at 3000 g and the aliquoted plasma was stored at − 80 ℃ for subsequent analysis. ELISA kits included the standard curve reagents (25 ng/mL top standard was serially diluted 1:1 to create the standard curve), the detection antibody (0.4 µg/mL), Streptavidin-HRP (Kingfisher Biotech Inc., MN, USA, catalogue number AR0068-001), 3,3’,5,5’-tetramethylbenzidine substrate solution (Kingfisher Biotech Inc., MN, USA, catalogue number AR0133-002) and a 0.18 M Sulfuric Acid stop solution. All plates were read at 450 nm (CLARIOstar^*Plus*^, BMG LABTECH, Australia). Samples were run in duplicates, and a coefficient of variability under 10% was classified as acceptable. No samples were re-run because they all fell within range of the standard curve.

### Bacterial blood count analysis

To quantify *E. coli* levels in the blood, a colony forming assay was used, as previously described [10]. Blood samples were serially diluted 1 in 10 in sterile 0.01 M PBS; 20 μL of undiluted and diluted blood were spotted onto Luria–Bertani agar plates and incubated in air overnight at 37 °C. Colonies were counted and expressed as CFU/mL.

### Statistical analysis

Data are expressed as mean ± standard error of the mean (SEM) after passing the tests for normality (D’Agostino and Pearson Omnibus test, confirmed by Kolmogorov–Smirnov test). Specific time-point comparisons within each group (splanchnic-denervated or sham) were performed using a paired Student t-tests with a Bonferroni correction of K = 2 to assess differences between baseline and 23-h and 30-h of bacteremia. An unpaired Student t-test was used to compare the *E. coli* (count) present between the sham and splanchnic-denervated group in the hepatic parenchyma and around hepatic vessels. A two-way repeated measures analysis of variance (ANOVA) was used to assess differences of MAP, HR, core body temperature, cytokines, bacterial counts, blood biochemistry, water consumption, food consumption and respiratory rate between splanchnic-denervated vs sham (Group), Time (from 24 to 30-h of bacteremia) and their interaction (GroupxTime). To correct for multiple comparisons a Šidák test was utilized (GraphPad Prism 8.0; GraphPad Software, La Jolla, CA). A two-sided P value < 0.05 was considered to be significant.

## Results

### Cardiovascular hemodynamics and febrile reponse to bacteremia and splanchnic denervation

There were no significant differences between the groups in the cardiovascular variables and body temperature over the 20-h baseline period.

**Fig. 2:**
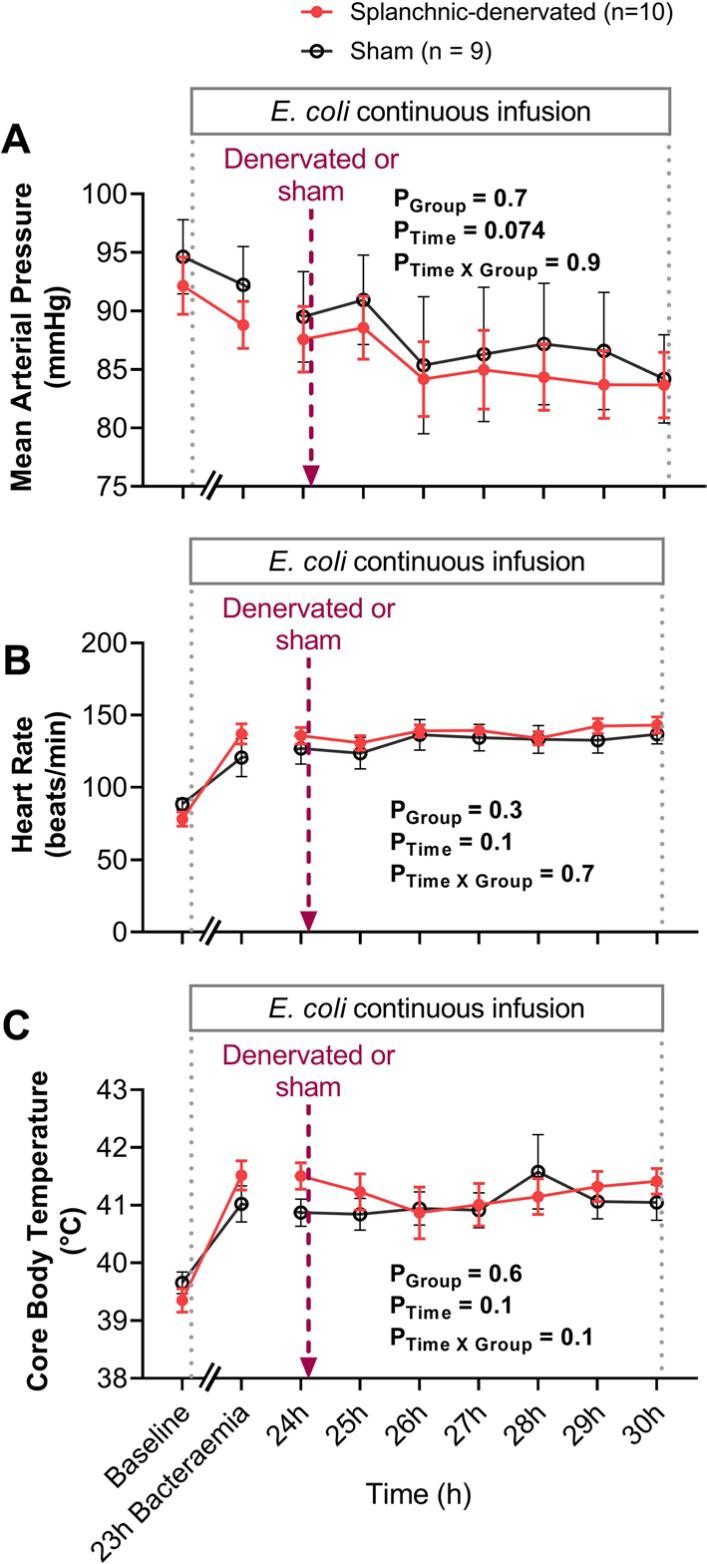
Changes in systemic hemodynamic in response to bilateral splanchnic denervation or sham procedure during ovine Gram-negative bacteremia. Mean arterial pressure (**A**), heart rate (**B**), and core body temperature (**C**) during infusion of *Escherichia coli* from 0 to 30-h. Animals were randomized to either splanchnic-denervated (n = 10) or untreated (sham, n = 9) at 24-h of bacteremia. Baseline is the mean of the 20th hour of the baseline period, and times 23 to 30-h of bacteremia are means of 1-h intervals. Data are presented as treatment group-specific mean ± SEM. P values represent the results of a 2-way ANOVA assessing differences between the groups (splanchnic-denervated vs. sham) and treatment time points (from 24 to 30-h of Gram-negative bacteremia) and the interaction between group and time. A two-sided P value < 0.05 was considered to be significant

In the sham group, MAP progressively declined from pre-morbid baseline levels until 30-h of bacteremia, but this overt effect was not statistically significant after adjusting for multiple comparisons (95 ± 3 to 84 ± 4 mmHg, P = 0.064) (unadjusted P = 0.032). There were no significant differences in the magnitude of the reduction in MAP between sham and splanchnic-denervated groups (Fig. 2A). Bacteremia was further characterized by increases in HR (89 ± 4 to 137 ± 7 beats/min, P < 0.001) and core body temperature (39.7 ± 0.2 to 41.0 ± 0.3 °C, P = 0.024) in the sham group by 30-h of *E. coli* infusion. The degree of tachycardia and increase in core temperature were not significantly different between treatment groups (Fig. 2B, C). The bacteremia-induced changes in arterial biochemistry were similar between the groups from 24-to-30-h of bacteremia (Table 1).

**Table 1:** Arterial blood gasses and lactate at 23-h of sepsis and following bilateral splanchnic nerve denervation or sham at 24-h of bacteremia in conscious sheep

	Baseline	23 h	Post-splanchnic/sham denervation		
			25 h	27 h	30 h
*Arterial pO*_*2*_*(mmHg)*					
SPX	104 ± 2	76 ± 7^++^	81 ± 5	79 ± 5	81 ± 6^++^
Sham	101 ± 3	79 ± 7^##^	79 ± 8	80 ± 7	77 ± 7^##^
*Arterial pCO*_*2*_ *(mmHg)*					
SPX	33 ± 1	29 ± 1	32 ± 1	32 ± 1	31 ± 1
Sham	32 ± 2	32 ± 2	32 ± 2	31 ± 2	32 ± 2
*Arterial Hb (g/dL)*					
SPX	11.4 ± 0.4	10.0 ± 0.4^+++^	9.4 ± 0.4	9.6 ± 0.3	9.2 ± 0.3^+++^
Sham	10.4 ± 0.5	9.4 ± 0.3	9.2 ± 0.5	9.2 ± 0.3	9.1 ± 0.3
*Arterial pH*					
SPX	7.51 ± 0.01	7.56 ± 0.01^++^	7.55 ± 0.01	7.56 ± 0.02	7.56 ± 0.01^+^
Sham	7.50 ± 0.01	7.56 ± 0.01^###^	7.57 ± 0.01	7.58 ± 0.01	7.57 ± 0.01^###^
*Arterial lactate (mmol/L)*					
SPX	0.52 ± 0.13	0.95 ± 0.09	0.87 ± 0.08	0.87 ± 0.15	0.78 ± 0.10
Sham	0.56 ± 0.09	1.17 ± 0.22	1.72 ± 0.78	1.03 ± 0.24	0.84 ± 0.15

*SPX*  Splanchnic-denervated (n = 10), *Sham* Sham-denervated (n = 9). ^*#*^*P* < *0.05, *^*##*^*P* < *0.01, *^*###*^*P* < *0.001* represent significant differences between baseline and 23-h and 30-h of Gram-negative bacteremia using a two-tailed paired Student T-test in the sham group (A Bonferroni correction of K = 2 was applied to p values). ^+^*P* < *0.05,* ^++^*P* < *0.01,* ^+++^*P* < *0.001* represent significant differences between baseline and 23-h and 30-h of Gram-negative bacteremia using a two-tailed paired Student T-test in the splanchnic-denervated group (A Bonferroni correction of K = 2 was applied to p values)

### Pro- and anti-inflammatory cytokine responses to bacteremia and splanchnic denervation

**Fig. 3:**
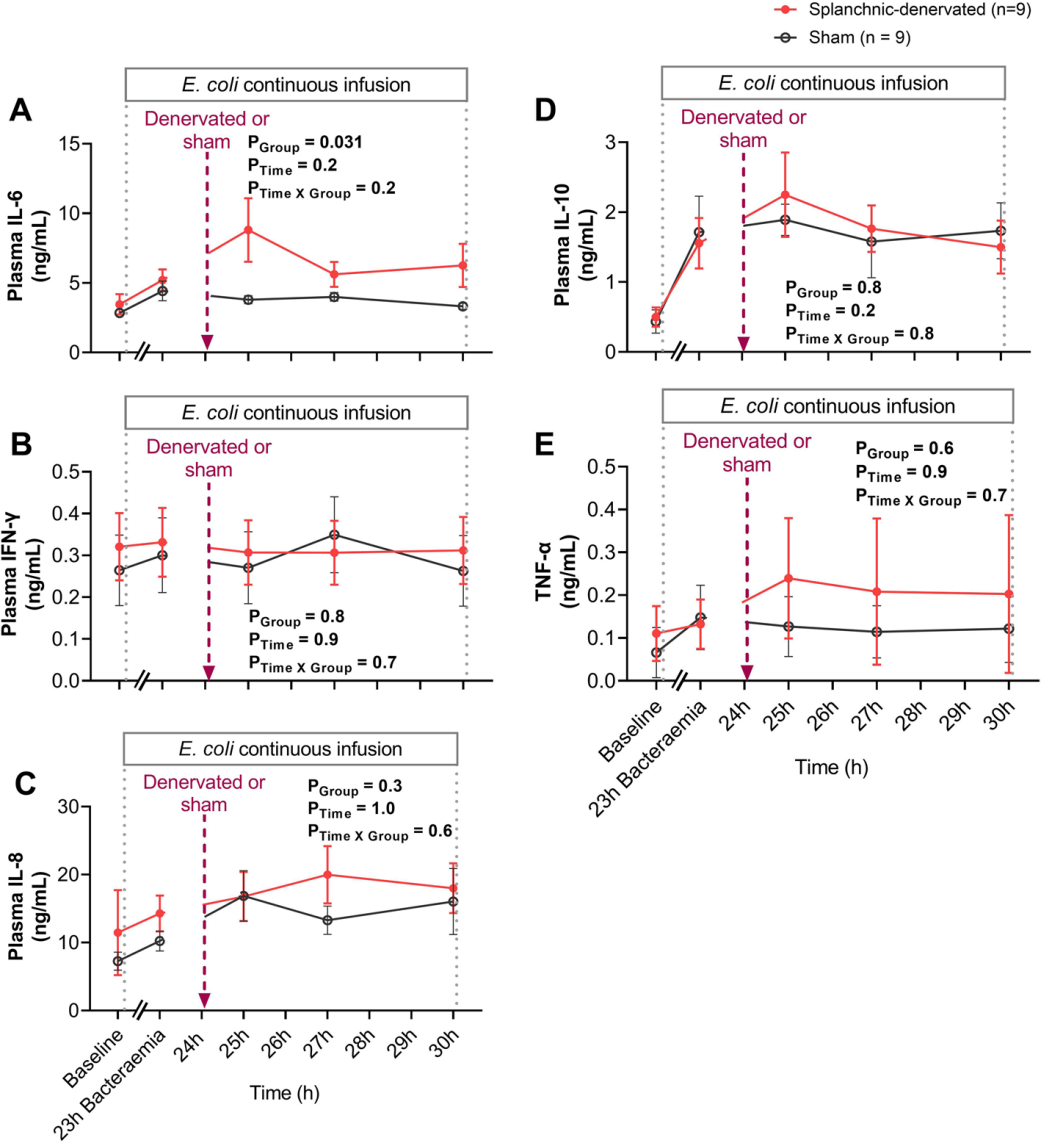
Changes in plasma inflammatory and anti-inflammatory cytokines in response to bilateral splanchnic denervation or sham procedure during ovine Gram-negative bacteremia. Plasma IL-6 (**A**), IFN-γ (**B**), IL-8 (**C**), IL-10 (**D**) and TNF-α (**E**) during infusion of *E. coli* from 0 to 30-h of bacteremia. Animals were randomized to either splanchnic-denervated (n = 9) or untreated (sham, n = 9) at 24-h of bacteremia. Baseline, 23-h, 25-h, 27-h and 30-h are the sampled timepoints. Data are presented as treatment group-specific mean ± SEM. P values represent the results of a 2-way ANOVA assessing differences between the groups (splanchnic-denervated vs sham) and treatment time points (from 24 to 30-h of bacteremia) and the interaction between group and time. A two-sided P value < 0.05 was considered to be significant

At 23-h of bacteremia, prior to splanchnic denervation, there were similar increases in the plasma concentrations of the pro-inflammatory cytokine, IL-6, in the sham (from 2.84 ± 0.23 to 4.42 ± 0.706 ng/mL, P = 0.049) and intervention groups, (3.46 ± 0.74 to 5.20 ± 0.76 ng/mL, P = 0.027). From 25-to-30-h of bacteremia, IL-6 levels remained consistently elevated in the sham group. In contrast, there was a further, transient elevation in plasma IL-6 levels following bilateral splanchnic denervation at 25-h of bacteremia (to 9.23 ± 2.54 ng/mL, P_Group_ = 0.031), before declining to levels that were similar to those in the sham treatment group by 30-h of bacteremia (to 6.26 ± 1.55 ng/mL) (Fig. 3A).

At 23-h of bacteremia, there was also an increase in the plasma concentrations of the anti-inflammatory cytokine, IL-10, in both the sham (from 0.44 ± 0.17 to 1.72 ± 0.51 ng/mL, P = 0.014) and splanchnic-denervated groups (from 0.50 ± 0.14 to 1.56 ± 0.36 ng/mL, P = 0.033) (Fig. 3D). These plasma concentrations of IL-10 remained similarly elevated in the sham and splanchnic-denervated groups from 25-to-30-h of bacteremia (Fig. 3D). There were also no significant differences between the sham and splanchnic-denervated groups in plasma concentrations of TNF-α, IL-8 and IFN-γ by 23-h of bacteremia or after intervention from 24-to-30-h (Fig. 3B, C, E).

### Effects of bacteremia and splanchnic denervation on blood bacterial counts

**Fig. 4:**
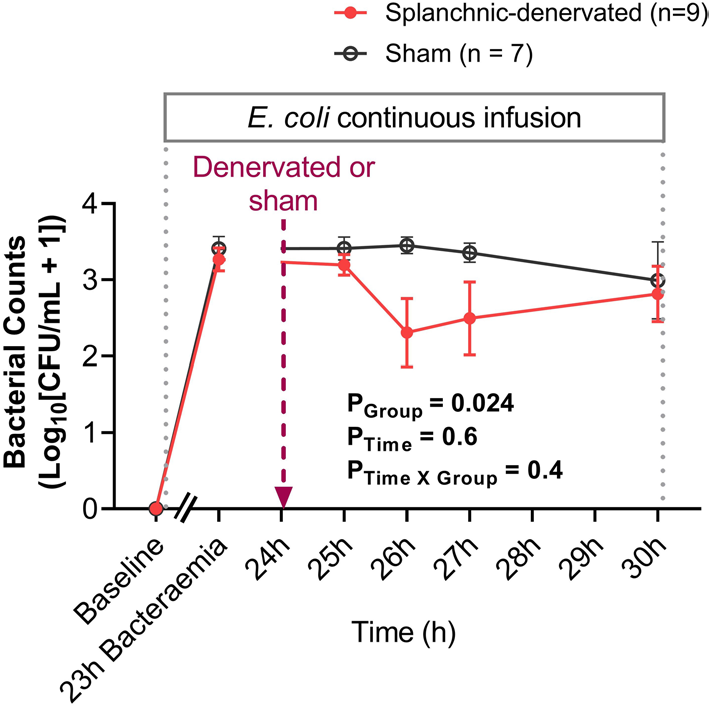
Changes in blood bacterial counts in response to bilateral splanchnic denervation or sham procedure during ovine Gram-negative bacteremia. Animals were randomized to either splanchnic-denervated (n = 9) or untreated (sham, n = 7) at 24-h of bacteremia. Baseline, 23-h, 25-h, 26-h, 27-h and 30-h are the sampling timepoints. Data are presented as treatment group-specific mean ± SEM. P values represent the results of a 2-way ANOVA assessing differences between the groups (splanchnic-denervated vs. sham) and treatment time points (from 24 to 30-h of bacteremia) and the interaction between group and time. A two-sided P value < 0.05 was considered to be significant

There were significant elevations in blood *E. coli* counts from undetectable levels at baseline to 23-h of bacteremia in the sham (to 3.41 ± 0.16 log_10_[CFU/mL + 1], P < 0.001) and splanchnic denervation groups (to 3.27 ± 0.15 log_10_[CFU/mL + 1], P < 0.001), prior to treatment allocation (Fig. 4). Blood bacterial counts remained elevated in the sham group in response to a continuous infusion of *E. coli* for 30-h (to 2.99 ± 0.51 log_10_[CFU/mL + 1]). In contrast, there was a transient reduction in blood *E. coli* counts in the splanchnic-denervated group by 26-h of bacteremia (2.31 ± 0.45 log_10_[CFU/mL + 1]; P_Group_ = 0.024), before returning to sham treatment levels by 30-h of *E. coli* infusion (to 2.82 ± 0.36 log_10_[CFU/mL + 1]; Fig. 4).

### Effects of bacteremia and splanchnic denervation on clinical state

**Fig. 5:**
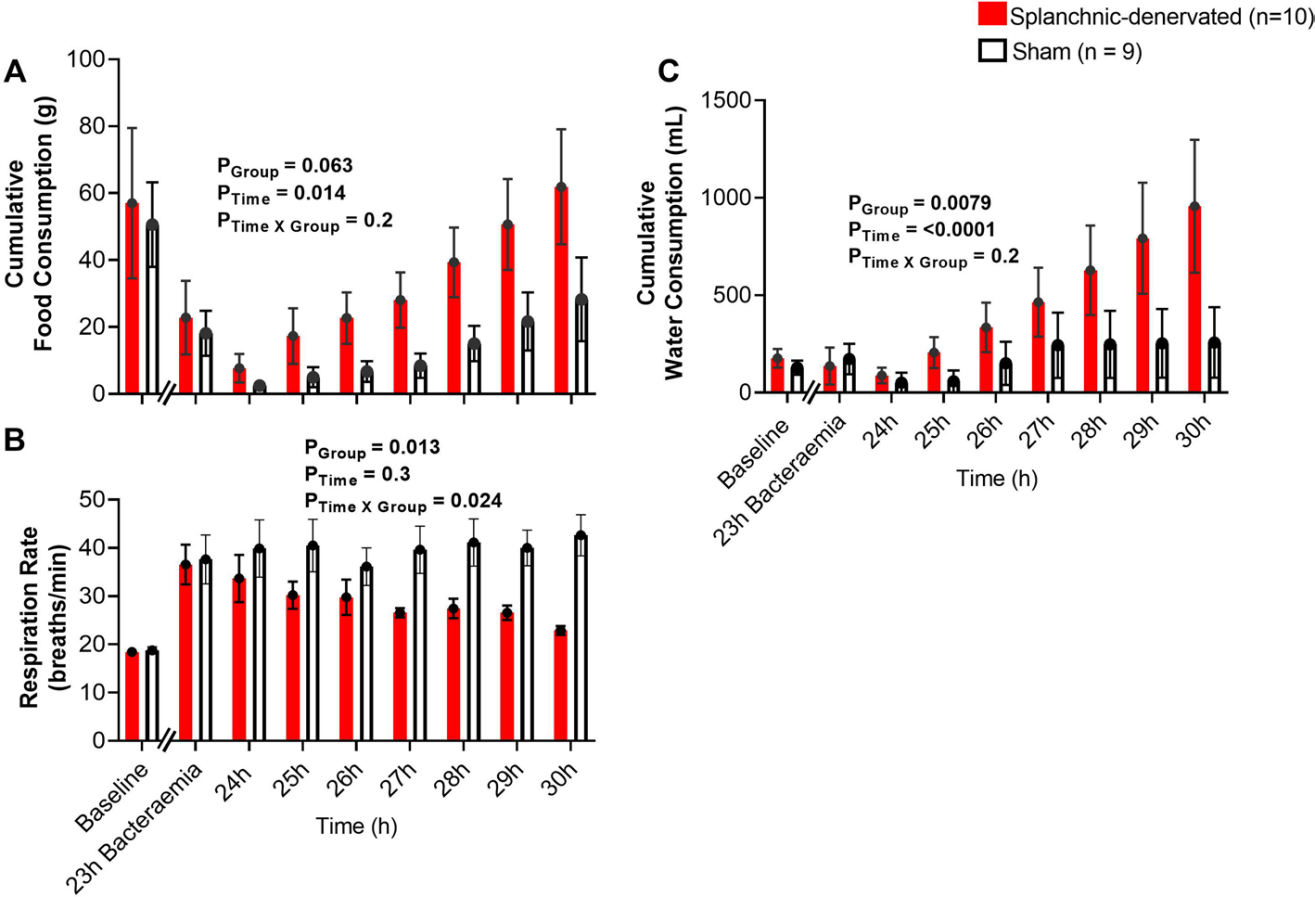
Changes in clinical status in response to bilateral splanchnic denervation or sham during ovine Gram-negative bacteremia. Cumulative food consumption (**A**), respiration rate (**B**) and cumulative water consumption (**C**), during infusion of *Escherichia coli* from 0 to 30-h. Animals were randomized to either splanchnic-denervated (n = 10) or untreated (sham, n = 9) at 24-h of bacteremia. Baseline is the average of the 20th hour of the baseline period, and times 23 to 30-h of bacteremia are means of 1-h intervals. Data are presented as treatment group-specific mean ± SEM. P values represent the results of a 2-way ANOVA assessing differences between the groups (splanchnic-denervated vs. sham) and treatment time points (from 24 to 30-h of Gram-negative bacteremia) and the interaction between group and time. A two-sided P value < 0.05 was considered to be significant

Splanchnic denervation-associated improvements in clinical state were accompanied by significant elevations in cumulative water intake from 24 to 30-h of bacteraemia (P_Group_ = 0.008). Cumulative food intake also showed a tendency to be higher in splanchnic-denervated sheep compared with sham treatment, but this overt effect did not reach statistical significance (P_Group_ = 0.063) (Fig. 5A, C). Respiratory rate was elevated by 23-h of bacteraemia in both groups prior to splanchnic denervation (18 ± 1 to 37 ± 4 breaths/min; P = 0.001) or sham treatment (19 ± 1 to 38 ± 5 breaths/min; P = 0.008) compared with pre-morbid baseline levels. The degree of tachypnoea was significantly reduced following splanchnic denervation (to 23 ± 1 breaths/min) compared with sham treatment (to 43 ± 4 breaths/min) by 30-h of bacteraemia (P_Group_ = 0.024) (Fig. 5B).

### Effect of splanchnic denervation on the localization of E. coli in the liver

**Fig. 6:**
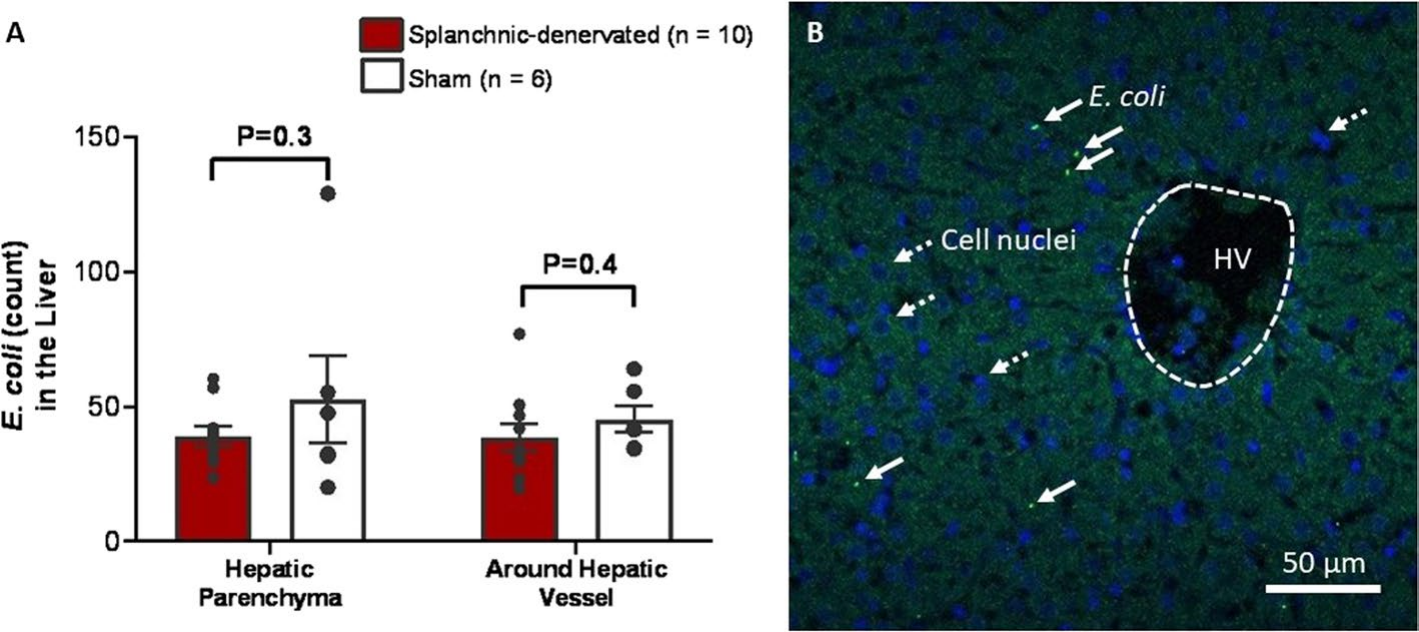
Counts of *E. coli* in the liver of sham and splanchnic-denervated sheep after 30-h of bacteremia. **A** Numbers of *E. coli* in hepatic parenchyma or around hepatic vessels in sham and splanchnic-denervated animals (n/group; NS two-tailed unpaired Student T test). **B** Representative image of hepatic parenchyma surrounding hepatic vessels (HV) taken at × 40 magnification showing the localization of *E. coli* (green) and cell nuclei are stained with DAPI (blue). White solid arrows indicate *E. coli* and white dashed arrows indicate the cell nuclei. Scale bar 50 µm

*E. coli* were found within the liver parenchyma of both experimental groups at post-mortem after 30-h of bacteremia. There were no significant differences between the sham and splanchnic-denervated groups in the numbers of *E. coli* present within the hepatic parenchyma (52.7 ± 16.1 vs 39.1 ± 3.7; P = 0.3) and around the hepatic vessels (45.3 ± 4.9 vs 38.6 ± 5.3; P = 0.4) (Fig. 6).

## Discussion

### Key findings

In a large mammalian model of live Gram-negative bacteremia, we determined the effect of bilaterally denervating the splanchnic sympathetic nerves, at a clinically appropriate interventional time-point of established infection. We found that splanchnic denervation transiently increased plasma levels of the pro-inflammatory cytokine, IL-6, and this was followed by a transient improvement in bacterial clearance and a sustained improvement in cumulative water intake and respiratory rate. Moreover, we demonstrated that splanchnic denervation did not exacerbate bacteremia-induced reductions in blood pressure. Finally, we found that this intervention did not change the level of sequestration of bacteria in the liver.

### Relationship to previous studies

In sepsis, a protracted sympathetic overstimulation has been proposed to contribute to the decreased vasopressor responsiveness to endogenous catecholamines and exogenously infused vasopressor agents, leading to severe hypotension and reduced organ perfusion [17]. Supporting this notion, we have previously reported that centrally acting sympatholytics, dexmedetomidine and clonidine, improve blood pressure management and sensitivity to catecholamine and non-catecholamine vasopressors (noradrenaline, phenylephrine, and angiotensin II) in established ovine Gram-negative sepsis [18, 19]. Similarly, clinical studies in human sepsis have reported that treatment with dexmedetomidine results in lower vasopressor requirements to maintain target MAP [20, 21]. These studies indicate that sympatho-modulatory interventions can confer a beneficial role on cardiovascular function in established infections. However, the use of non-selective sympatholytic drugs including clonidine and dexmedetomidine attenuated the increase in plasma IL-6 and enhanced the increase in IL-10 levels in ovine sepsis, likely due to activation of anti-inflammatory cholinergic pathways [19, 22]. Collectively, these studies provided the rationale to selectively target the splanchnic sympathetic nerves to demonstrate the safety and effectiveness of such an intervention in a non-hypotensive ovine model of bacteremia.

The splanchnic sympathetic nerves are implicated as the efferent arm of a negative feedback mechanism whereby the brain inhibits innate immune responses to inflammatory stimuli [8–10, 23–28]. We previously reported that in rats made endotoxemic with intravenous lipopolysaccharide (LPS), prior section of the splanchnic sympathetic nerves resulted in enhanced plasma levels of pro-inflammatory cytokines (TNF-α, IL-6 and IFN-γ) and a reduction in the anti-inflammatory cytokine, IL-10 [27]. Subsequently we reported that sheep with previously sectioned splanchnic nerves mounted an exaggerated inflammatory cytokine response to an intravenous bolus injection of live *E. coli* and rapidly cleared the bacteria from their bloodstream [10].

### Cytokine responses

In the current study, we show that, sectioning the splanchnic nerves during established Gram-negative bacteraemia, enhances IL-6 levels, albeit transiently. These responses demonstrate a positive biological signal of splanchnic denervation when compared with our previous findings that plasma IL-6 levels temporally declined from 24 to 32-h of sepsis in sheep with established hypotensive sepsis [19, 29, 30]. Moreover, these enhanced IL-6 responses were followed by accelerated bacterial clearance and improved clinical state, despite no treatment with antibiotics. IL-6 is an important contributor to the pro-inflammatory response to infections that remains elevated up to 32-h of sepsis [31, 32]. This contrasts with plasma levels of other inflammatory cytokines including TNF-α and IFN-γ that rise transiently in early stages of infection and then fall towards baseline levels at later stages of sepsis [10, 33, 34]. It is therefore not surprising that we observed no detectable change in plasma TNF-α and IFN- γ by 23-h of bacteraemia, nor any response of those cytokines to splanchnic nerve denervation.

### Bacterial clearance

The transient elevation in IL-6 induced by splanchnic denervation was followed by an expedited clearance of bacteria from the bloodstream until 3-h post splanchnic denervation. Monocytes, macrophages, and neutrophils contain IL-6 receptors, and their function is regulated by IL-6 signaling pathways [32]. At the onset of the innate immune response to a pathogen entering the bloodstream, neutrophils are the primary phagocytes involved in limiting the systemic dissemination of the infection. IL-6 plays an important role in modulating the magnitude of the neutrophil response [35, 36]. As the inflammatory response continues, monocytes and macrophages are recruited to assist [31] and IL-6 is an important regulator in this transition, ensuring that inflammation is sufficient to continue mounting an adequate response to a persisting infection [35, 36]. In the current study, the elevated IL-6 levels after splanchnic-nerve denervation may have stimulated these innate immune cells to expedite the clearance of *E coli* from the blood. The inability to assess the direct interaction between IL-6 and neutrophil function is a limitation of the current experimental design that warrants further investigation following either non-pharmacological or pharmacological inhibition of the splanchnic sympathetic nerves in future experiments.

Interestingly, splanchnic denervation at 24-h of bacteremia did not significantly affect IL-10 levels. Differential regulation of IL-6 and IL-10 responses to splanchnic nerve denervation aligns with our recent findings in rodent endotoxemia. We recently found that distinct splanchnic sympathetic efferent pathways regulate pro- and anti-inflammatory cytokines in response to inflammatory stimuli [37]. The neural-driven adrenaline, acting via β_2_ adrenoreceptors, regulates IL-10 responses. In contrast, the increase in TNF-α, which stimulates the subsequent IL-6 release in response to endotoxemia, is restrained by the splanchnic nerves that provide neural control to abdominal organs including the spleen and involves non-β_2-_ adrenoreceptor mechanisms [37].

### Clinical state

Following splanchnic sympathetic denervation, there was a sustained improvement in clinical status, despite blood bacterial counts returning to sham treatment levels by 30-h. The liver plays a vital role in the clearance of pathogens entering the bloodstream to mitigate the spread of bacteria to more susceptible organs including the brain and kidneys [14, 38, 39]. Accordingly, the similarities in blood bacterial counts by 30-h of infection were confirmed by our immunohistochemical analysis of the liver tissue, which demonstrated similar numbers of *E. coli* present in the hepatic parenchyma and hepatic vessels in both groups. The restoration of IL-6 and bacterial counts in denervated animals to sham levels by the end of the experiment (30-h) is likely due to the continued infusion of live *E. coli* outstripping the enhanced bacterial clearance. It is conceivable that the initiation in improvement in clinical state could be attributed to an enhanced immune response and expedited bacterial clearance, which were evident until 27-h of bacteraemia. It is also important to note that snaring the splanchnic nerves will destroy not only the efferent but also the afferent fibres that mediate visceral pain and febrile signalling induced by the bacteraemia [40–42]. This may have contributed to the sustained clinical improvement after splanchnic denervation, despite a rebound in bacterial counts. Future studies are therefore warranted to test the effect of splanchnic nerve section on blood pressure and bacterial clearance in more severe models of Gram-negative sepsis-induced hypotension, without further *E. coli* infusion and/or with antibiotic treatment, which may better mimic patients with sepsis after removal of the infection source in ICUs [43].

## Conclusions

Our findings demonstrate that interrupting splanchnic nerve activity in established bacteremia is safe for blood pressure control, increases circulating IL-6 levels, expedites the clearance of bacteria from the bloodstream and improves the clinical state. Although the enhanced bacterial clearance was transit in the face of a continuous intravenous infusion of bacteria, such an intervention may prove more effective in cases where the infection source is removed. Importantly, our findings show that the neural reflex persists for at least 24-h into established bacteremia, and that its sustained anti-inflammatory action may hamper the ability of innate immune mechanisms to overcome an infection. Future studies are warranted to explore the effects of pharmacological and other means, such as acute nerve block [44], to inhibit splanchnic sympathetic nerve activity in the setting of severe bacterial infection.

### Supplementary Information

**Additional file 1: Table S1.** List of enzyme-linked immunosorbent assay reagents. **Figure S1.** Silk snare, cannula for infusion and outer tube surgically implanted bilaterally on splanchnic sympathetic nerves.

## Data Availability

Data available upon request due to ethical reasons.

## Abbreviations

SNA: Sympathetic nerve activity
MAP: Mean arterial pressure
*E.**coli*: *Escherichia coli*
i.v.: Intravenous
HR: Heart rate
CFU: Colony forming units
IL-6: Interleukin-6
IFN-γ: Interferon-gamma
IL-8: Interleukin-8
IL-10: Interleukin-10
TNF-α: Tumour necrosis factor-alpha
ELISA: Enzyme-linked immunosorbent assay
SEM: Standard error of the mean
ANOVA: Analysis of variance
LPS: Lipopolysaccharide
