# Supplementary material for: Splanchnic sympathetic nerve denervation improves bacterial clearance and clinical recovery in established ovine Gram-negative bacteremia
Source: Intensive Care Med Exp. 2023 Aug 3;11:53. doi: 10.1186/s40635-023-00530-6 (PMC10400745; doi:10.1186/s40635-023-00530-6)
Supplement: Supplementary file 1 — Additional file 1: Table S1. List of enzyme-linked immunosorbent assay reagents. Figure S1. Silk snare, cannula for infusion and outer tube surgically implanted bilaterally on splanchnic sympathetic nerves. [file 40635_2023_530_MOESM1_ESM.docx]

**Online Additional file**

Splanchnic sympathetic nerve denervation improves bacterial clearance and clinical recovery in established ovine Gram-negative bacteremia

Rachel M. Peiris^1^, Clive N. May^1,2^, Lindsea C. Booth^1^, Robin M. McAllen^1^, Michael J. McKinley^1^, Sally Hood^1^, Davide Martelli^3^, Rinaldo Bellomo^2,4,5^ and Yugeesh R Lankadeva^1,2^

^1.^ Preclinical Critical Care Unit, Florey Institute of Neuroscience and Mental Health, University of Melbourne, Victoria, Australia.

^2.^ Department of Critical Care, Melbourne Medical School, University of Melbourne, Victoria, Australia.

^3.^ Department of Biomedical and Neuromotor Sciences, University of Bologna, Bologna, Italy.

^4.^ Department of Intensive Care, Austin Health, Victoria, Australia.

^5.^ Australian and Intensive Care Research Centre, Monash University, Melbourne, Australia

| **Enzyme-linked immunosorbent assay** | **Capture Antibody** | **Detection Antibody** | **Standard Protein** |
| --- | --- | --- | --- |
| **Ovine Interferon (IFN)γ** | Anti-Ovine IFNγ Polyclonal Antibody (Catalogue number KP1811V-100) | Biotinylated Anti-Ovine IFNγ Polyclonal Antibody (Catalogue number KPB1812V-050) | Ovine IFNγ Recombinant Protein (Catalogue number RP0491V-005) |
| **Ovine Tumour Necrosis Factor (TNF)-α** | Anti-Ovine TNF-α Polyclonal Antibody (Catalogue number KP1454V-100) | Biotinylated Anti-Ovine TNF-α Polyclonal Antibody (Catalogue number KPB1455V-050) | Recombinant Ovine TNF-α (Catalogue number RP0902V-005) |
| **Ovine Interleukin (IL)-6** | Anti-Bovine IL-6 Polyclonal Antibody (Catalogue number KP0652B-100) | Biotinylated Anti-Bovine IL-6 Polyclonal Antibody (Catalogue number KPB0653B-050) | Recombinant Ovine IL-6 (Catalogue number RP0367V-005) |
| **Bovine IL-10** | Anti-Bovine IL-10 Polyclonal Antibody (Catalogue number KP1255B-100) | Biotinylated Anti-Bovine IL-10 Polyclonal Antibody (Catalogue number KPB1256B-050) | Recombinant Bovine IL-10 (Catalogue number RP0379B-005) |
| **Swine IL-8** | Anti-Swine IL-8 Polyclonal Antibody  (Catalogue number PB0143S-100) | Biotinylated Anti-Swine IL-8 Polyclonal Antibody  (Catalogue number PBB0266S-050) | Recombinant Swine IL-8  (Catalogue number RP0109S-005) |
| **Table S1:** List of enzyme-linked immunosorbent assay reagents | | | |


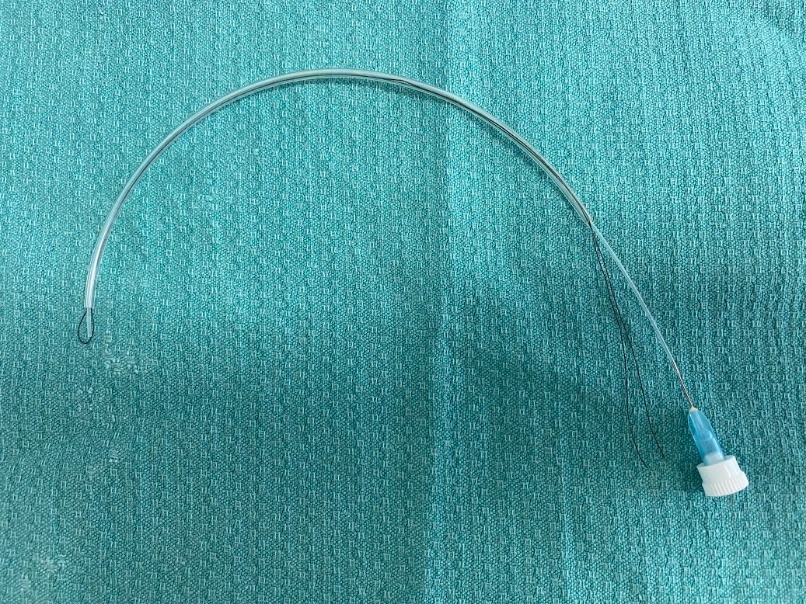


**Polyethylene cannula for infusion of local anaesthetic**

**Silk Snare**

**Outer Vinyl Tube**

**Figure S1:** Silk snare, cannula for infusion and outer tube surgically implanted bilaterally on splanchnic sympathetic nerves
